# A cell atlas of multiple liver organoids and the fetal liver based on scRNA-seq

**DOI:** 10.1016/j.isci.2026.114955

**Published:** 2026-02-07

**Authors:** Qinfeng Ma, Xu Zhang, Jianbo Pan

**Affiliations:** 1 Basic Medicine Research and Innovation Center for Novel Target and Therapeutic Intervention, Ministry of Education, College of Pharmacy, and the Reproductive Medicine Center, the First Affiliated Hospital, Chongqing Medical University, Chongqing 400016, China; 2 Chongqing Blood Center, Chongqing 400015, China; 3 Precision Medicine Center, the Second Affiliated Hospital of Chongqing Medical University, Chongqing 400010, China; 4 Reproductive Medicine Center, the First Affiliated Hospital, Chongqing Medical University, Chongqing 400016, China

**Keywords:** Medicine, Integrative aspects of cell biology, Transcriptomics

## Abstract

Understanding the cellular characteristics and regulatory networks of cells during the growth of human liver organoids is crucial for comprehending liver development and function. However, a comprehensive cell atlas of liver organoids spanning multiple culture protocols is still lacking. To address this gap, we integrated scRNA-seq datasets of liver organoids from public repositories with published human fetal liver datasets, constructing a unified liver organoid cell atlas comprising 217,025 high-quality cells. This atlas captures diverse cellular compositions across culture conditions of liver organoids. Comparative analyses revealed deficiencies in hematopoietic lineage representation in organoids relative to fetal tissues. Subpopulation and pseudotime analyses provided insights into hepatoblast fate transitions, while cell-cell communication analysis highlighted prominent NOTCH, VEGF, HGF, and WNT signaling activities in organoids. Together, our study delineates differences between liver organoids and fetal tissues and enables systematic comparison across culture protocols, providing a framework for protocol evaluation and optimization.

## Highlights

• Single-cell transcriptomic atlas of liver organoids was constructed across culture protocols
• A systematic comparison was performed among protocols and between organoids and fetal liver
• Cell trajectory and communication were analyzed to guide culture system optimization

## Graphical abstract

**Figure:**
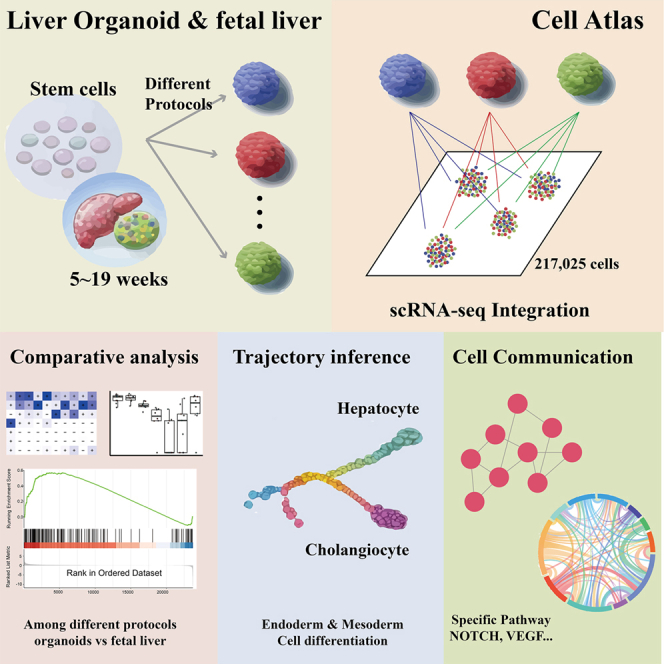

## Introduction

The liver, a central metabolic organ in the human body, plays essential roles in the synthesis and metabolism of carbohydrates, lipids, and proteins, as well as in the systemic transport of hormones, drugs, and other substances. Additionally, it functions as a specialized immune organ capable of effectively clearing exogenous substances and pathogenic microorganisms from the blood.^1^ Moreover, during development, the liver performs hematopoietic functions and synthesizes coagulation factors.^2^ Liver development is initiated by the directed differentiation of the foregut endoderm, where a subset of endodermal cells acquires a hepatic fate under the induction of signaling pathways such as the TGFβ signaling pathway, giving rise to the liver bud (hepatic diverticulum).^3^ The cells of the diverticulum further differentiate into hepatoblasts, which proliferate and invade the septum transversum mesenchyme to form the liver primordium. Hepatoblasts possess bipotent differentiation capacity, giving rise to hepatocytes that form the functional metabolic units of the liver and cholangiocytes that establish the bile drainage network.^4^ Moreover, surrounding mesoderm-derived cells infiltrate the developing liver tissue, with a subset differentiating into endothelial cells to form the hepatic sinusoidal network. The remaining cells differentiate into mesenchymal cells, which further give rise to hepatic stellate cells and mesothelial cells, collectively contributing to the supportive microenvironment of the liver.^5^

Given the critical role of the liver in human metabolism, the construction of *in vitro* models has significant value for drug discovery. Liver models cultivated using organoid technology can facilitate high-throughput testing of drug efficacy and toxicity, thereby enhancing predictive capabilities in preclinical drug screening.^6^^,^^7^^,^^8^ Currently, liver organoid culture relies primarily on two stem cell sources: pluripotent stem cells (PSCs) and adult stem cells (ASCs). PSC-derived liver organoids typically undergo three developmental stages: directed endoderm induction, hepatic lineage specification, and organoid maturation. Specifically, PSCs are first induced to differentiate toward the definitive endoderm via the synergistic action of growth factors such as activin A, BMP4, FGF4, and CHIR99021. On day 6, the cell aggregates are embedded in Matrigel and cultured with growth factors promoting the FGF and EGF signaling pathways to encourage the self-organization of the organoids. Four days later, the base medium is changed to hepatocyte culture medium (HCM), supplemented with HGF, dexamethasone, and oncostatin M (OSM) to drive the maturation of liver-specific functions.^9^ In contrast, ASC-derived organoids do not require an endoderm induction step.^10^ Adult stem cells isolated from liver tissue, such as cholangiocyte progenitor cells, can form three-dimensional structures in medium containing EGF, R-spondin 1 (RSPO1), FGF, HGF, and BMP.^11^^,^^12^ This cultivation system can be used to produce two subtypes of organoids: bile duct organoids (BDOs) and human liver organoids (HLOs). BDOs express high levels of cholangiocyte markers (KRT19 and KRT7) and possess bile acid transport capabilities, whereas HLOs are enriched in hepatocyte-specific genes (HNF4A and AFP) and display functional traits such as urea synthesis and drug metabolism. The difference between these two organoid types arises from the intrinsic state of the ASCs and the specific combination of inductive factors in the culture environment.

Liver organoids offer promising *in vitro* models for drug metabolism, toxicity testing, and disease modeling. Single-cell transcriptomics analysis enables detailed characterization of cellular and molecular features, guiding the optimization of culture conditions. However, challenges remain, including inconsistent protocols, variability in cell composition and maturity, and a lack of systematic cross-study comparisons. To address these issues, we integrated single-cell transcriptomic data from multiple studies via OrganoidDB^13^ to construct a comprehensive liver organoid cell atlas. This atlas reveals differences across protocols and highlights distinctions from fetal liver tissue, providing a foundation for standardizing and improving liver organoid culture systems.

## Results

### Cellular diversity of liver organoids under different protocols

**Figure 1:**
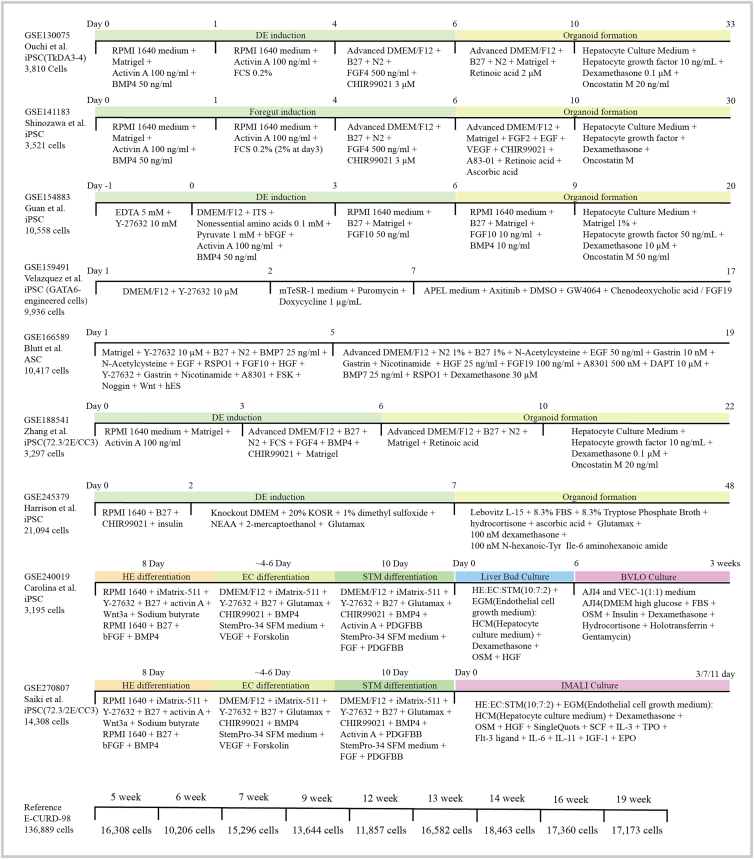
Schematic view of the liver organoid culture protocols and fetal liver information The diagram illustrates the major differentiation strategies used to generate liver organoids from pluripotent or adult stem cell sources. It summarizes key stages of the protocols, including the growth factors and small molecule compounds used in each stage, as well as the corresponding culture durations. In addition, the schematic includes information on fetal liver samples and indicates the number of cell samples from each protocol that were included in the present analysis.

**Figure 2:**
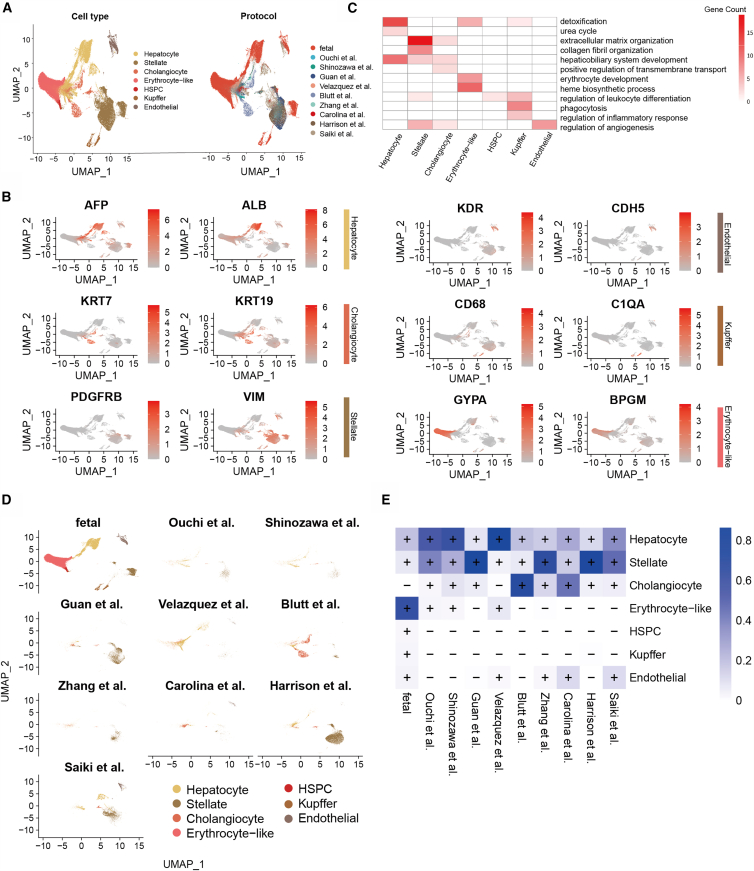
Overview of the liver organoid cell atlas (A) UMAP visualization of 217,025 high-quality single cells integrated from liver organoid and fetal liver datasets, colored by annotated cell type (left) and by culture protocol (right). (B) UMAP-based heatmap showing the expression patterns of representative marker genes for major liver-associated cell types, including hepatocytes, cholangiocytes, hepatic stellate cells, endothelial cells, Kupffer cells, and erythrocyte-like cells. (C) Heatmap of Gene Ontology (GO) terms enriched in cell type-specific gene sets, with color intensity indicating the number of genes associated with each functional term. (D) UMAP visualization of cells colored by cell type, illustrating how cells derived from different experimental conditions are distributed within the integrated transcriptional landscape. (E) Heatmap summarizes the presence and relative abundance of each cell type across the different organoid culture protocols; cell types accounting for more than 10% of the cells in a given protocol are indicated by a plus sign.

In this study, we integrated single-cell transcriptomic data from 15 liver organoid samples across 9 independent studies (Figure 1), along with human fetal liver data spanning 5–19 weeks of gestation, to systematically compare differences across culture protocols and between organoids and native embryonic tissue. After standardized quality control and filtering of low-quality cells, a total of 217,025 cells were analyzed, including 80,136 from organoids and 136,889 from fetal liver tissue. Based on cell type-specific marker genes and GO-based annotations, all cells were classified into seven major cell types: hepatocytes, cholangiocytes, stellate cells, endothelial cells, hematopoietic stem/progenitor cells (HSPCs), erythrocyte-like cells, and Kupffer cells (Figures 2A, 2B, 2C, and S1). In summary, liver organoids derived from diverse protocols have broadly comparable cellular lineage compositions (Figure 2D).

Most differentiation protocols follow a stepwise approach involving definitive endoderm induction, hepatic specification, and organoid maturation (e.g., Ouchi et al., Shinozawa et al., Guan et al., and Zhang et al.)^6^^,^^14^^,^^15^^,^^16^ to generate multicellular liver-like structures. The cell type proportions were calculated for each experimental condition (Figure 2E). Notably, within this group of comparable protocols, the organoids from Guan et al. presented a high abundance of stellate cells (∼85%), potentially due to the absence of retinoic acid (RA) in the culture. RA is known to direct progenitor cells toward the hepatocyte lineage during liver development and promote regeneration after injury. RA-deficient embryos exhibit disorganized mesoderm surrounding the liver bud, supporting its critical role.^17^^,^^18^^,^^19^ In organoids derived from ASCs, such as those generated by the Blutt^10^ group, cholangiocytes accounted for ∼72% of the population, which is consistent with the intrinsic biliary differentiation propensity of ASCs. In contrast, Velazquez et al.^20^ engineered iPSCs overexpressing key regulators (GATA6, PROX1, and ATF5), resulting in a hepatocyte-enriched organoid (∼86%) and the unexpected emergence of erythroid-like cells (7%) and endothelial cells (1.7%), suggesting partial activation of hematopoietic niche components. Vascularized organoid protocols from Carolina^21^ and Saiki^22^ yielded substantial endothelial cell populations (∼11%), whereas Harrison et al.’s^23^ model generated a large fraction of stellate cells (∼83%). Given that both stellate and endothelial cells originate from mesodermal precursors during liver development, these observations raise the possibility that mesenchymal populations within organoids may retain some degree of endothelial differentiation potential.

Overall, despite considerable heterogeneity among protocols, current liver organoids recapitulate key aspects of fetal liver cellular diversity and partially mimic hepatic functionality. Importantly, these observations are supported by analyses performed after systematic batch-effect correction across datasets, as well as UMAP visualizations stratified by selected experimental factors (Figure S2), which indicate that the observed cellular patterns are not driven solely by technical variation. Together, these results support their utility as *in vitro* platforms for drug metabolism studies and developmental research.

### Comparison of liver organoids and the fetal liver

Hematopoietic stem and progenitor cells (HSPCs) begin migrating to the liver bud around gestational week 6, at which point the fetal liver becomes the primary site of hematopoiesis and serves as a supportive niche for HSPC proliferation and differentiation.^24^^,^^25^^,^^26^ In fetal liver samples, HSPCs and erythroid-like cells begin to emerge at week 5 and markedly increased in abundance by week 7, reflecting the central role of the liver in embryonic hematopoiesis.^27^ In contrast, organoid samples present a minimal presence of hematopoietic cell types (average proportion ∼1.5%) and display an absence of functional Kupffer cells (Figure 2E), suggesting that current liver organoid systems may not fully recreate the hematopoietic microenvironment and may exhibit limited hematopoietic functionality. Notably, recent work by Li et al. supports this view. They found that the liver organoids generated by traditional methods generally lack hematopoietic activity, and by co-culturing the liver organoids with erythro-myeloid progenitors (EMPs) derived from iPSCs, the hematopoietic process at the fetal liver stage was successfully simulated, and liver organoids containing functional Kupffer cells were obtained.^28^ Moreover, cholangiocytes are nearly absent from early fetal liver samples (<0.01%) (Figure 2E), which is consistent with the predominance of hepatocyte development during early liver organogenesis, when the biliary system remains underdeveloped.^29^^,^^30^ However, the proportion of cholangiocytes in liver organoids varies widely (0.5%–72%), underscoring the strong influence of culture conditions on biliary lineage specification.

**Figure 3:**
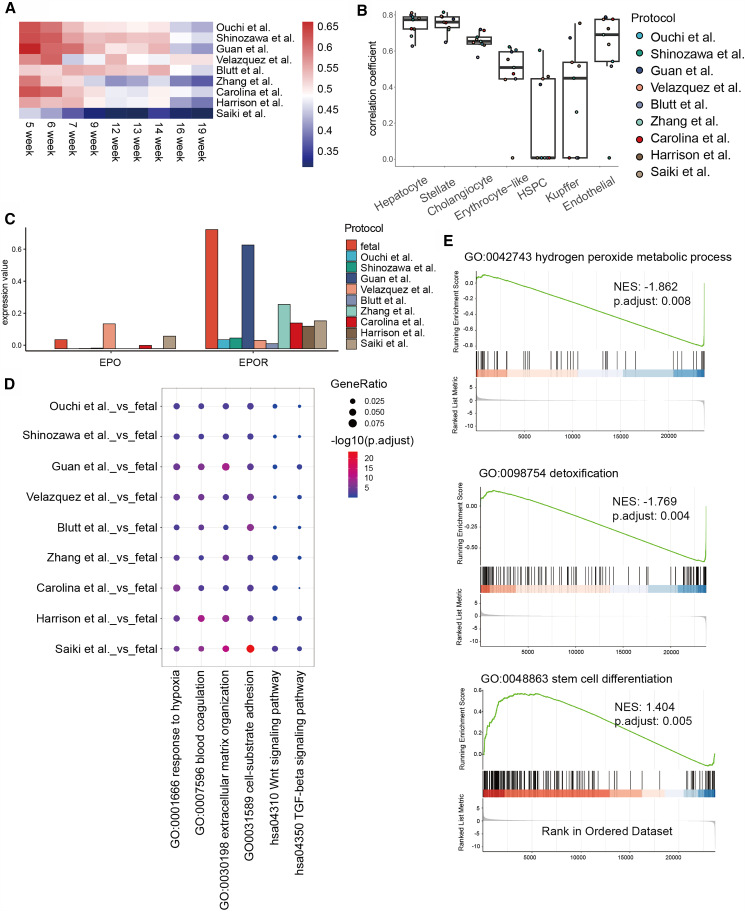
Comparative analysis of liver organoids and fetal tissues (A) Heatmap displays transcriptomic correlation coefficients between organoids generated under different culture protocols and fetal liver samples across distinct developmental time points, providing an overview of developmental-stage similarity. (B) Correlation analysis between corresponding cell types in each organoid protocol and their counterparts in fetal liver tissue. (C) Average expression levels of erythropoiesis-related genes across different organoid protocols and fetal liver samples. (D) GO and KEGG pathway enrichment analyses of genes upregulated in each organoid protocol relative to fetal liver tissue, and select specific pathways to display them using bubble charts to highlight biological processes and pathways preferentially represented in organoids. (E) GSEA compares global gene expression profiles between organoids and fetal liver tissue, summarizing systematic transcriptional differences at the pathway level.

We further compared the transcriptomic profiles of liver organoids and fetal tissues to evaluate the biological fidelity of the organoid models. First, we compared the transcriptional correlations between different protocols of organoids and embryonic samples at different developmental stages. These results showed that current culture protocols tend to generate liver organoids whose transcriptional profiles more closely resemble early-stage fetal liver (5–6 weeks), whereas their similarity to later developmental stages decrease substantially (Figures 3A and S3), this is consistent with the results of the previous report.^31^ Pearson’s correlation analysis of highly variable genes revealed strong transcriptional concordance between organoids and fetal tissues for hepatocytes, cholangiocytes, hepatic stellate cells, and endothelial cells (R > 0.6), suggesting that organoids effectively recapitulate the molecular features of core hepatic parenchymal and stromal cell types (Figure 3B). However, hematopoietic-related cell types are consistently poorly correlated because of their sparse representation in organoids, highlighting the limitations of current culture systems in modeling the hematopoietic niche of the liver. Notably, the expression levels of erythropoietin (EPO) and its receptor (EPOR) were significantly greater in fetal samples than in most organoid models, further supporting the absence of hematopoietic activity (Figure 3C).

We subsequently performed comparative analyses between organoids cultured under different protocols and fetal liver samples. By extracting the genes that showed an upregulated trend in the organoids (logFC >0.25), we performed an enrichment analysis and selected the significantly enriched pathways (*p* < 0.05). We observed that genes associated with the TGFβ and WNT signaling pathways were upregulated in the organoids (Figure 3D). These pathways are known to be involved in the maintenance of stemness, suggesting that organoid cells remain in an immature state with high developmental potential. In addition, genes related to extracellular matrix (ECM) organization were also upregulated in the organoids, reflecting the ongoing demand for matrix remodeling during *in vitro* three-dimensional culture. Gene set enrichment analysis (GSEA) comparing the overall transcriptomic profiles of the organoids and fetal samples further confirmed the significant activation of stem cell differentiation pathways in the organoids (Figure 3E). In contrast, pathways such as hydrogen peroxide metabolic processes were enriched in fetal samples, suggesting that organoids may reside in a comparatively more hypoxic microenvironment. From a functional perspective, these results also imply that the detoxification capacity of organoids may be substantially less developed than that observed in fetal liver tissues.

These findings suggest that while current liver organoids can recapitulate the molecular and genetic characteristics of hepatic parenchymal and biliary lineages, their functional maturity largely resembles the early stages of fetal liver development. The absence of hematopoietic activity, dysregulated metabolic pathways, and limited detoxification capacity underscore the limitations of current *in vitro* models in capturing the complex physiological functions of the liver. Moving forward, strategies such as vascularization to increase oxygen delivery, optimized growth factor combinations to promote metabolic maturation, and the integration of hematopoietic-supportive microenvironments will be essential to improve the utility of liver organoids in drug screening and disease modeling.

### Subpopulation analysis

**Figure 4:**
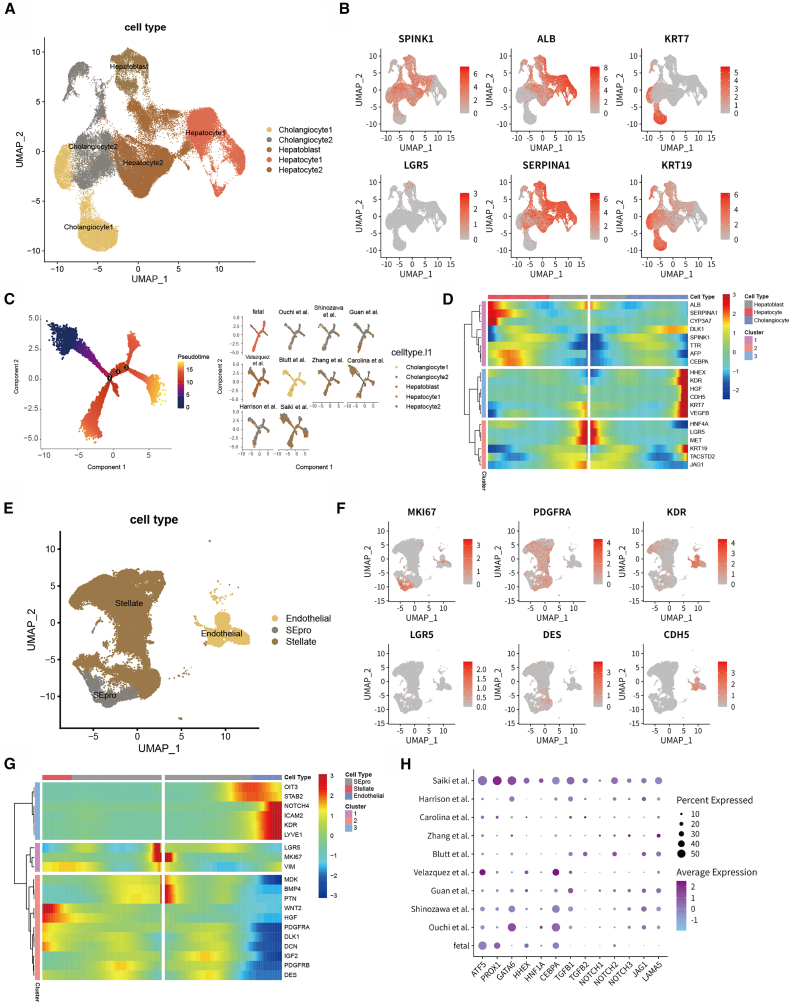
Subcluster analysis of endoderm- and mesoderm-derived cells (A–D) Subcluster analysis of endoderm-derived cells. (A) UMAP visualization of endoderm-derived cells extracted from the integrated dataset, with distinct colors indicating annotated cell types, including hepatoblasts, hepatocyte (hepatocyte1), cholangiocyte (cholangiocyte1), and two transitional cell types (hepatocyte2 and cholangiocyte2). (B) UMAP-based heatmap illustrates the expression patterns of representative marker genes specific to hepatoblasts, hepatocytes, and cholangiocytes. (C) Pseudotime-inferred developmental trajectories of endoderm-derived cells (left) and comparison of differentiation trajectories across different organoid culture protocols (right), with cells colored by annotated cell type. (D) Expression dynamics of fate-associated genes along the pseudotime trajectories downstream of branch node 1 identified in panel (C), illustrating branch-specific transcriptional changes associated with divergent lineage tendencies inferred from the developmental trajectory. (E–G) Subcluster analysis of mesoderm-derived cells. (E) UMAP visualization of mesoderm-derived cell lineages, with distinct colors denoting SEpro, hepatic stellate cells, and endothelial cells. (F) UMAP-based heatmap shows the expression of lineage-specific marker genes for SEpro, stellate cells, and endothelial cells. (G) Expression dynamics of fate-associated genes along pseudotime branches emerging from branch node 3 in Figure S4B, highlighting branch-dependent transcriptional patterns that are consistent with divergent lineage trajectories inferred from the developmental analysis. (H) Average expression levels of selected transcription factors and signaling pathway-related genes in endoderm-derived cells across different organoid culture protocols.

In this study, we systematically analyzed the subtypes and developmental regulation of endoderm- and mesoderm-derived cell populations in liver organoids. Reclustering of endoderm-derived cells (hepatocytes and cholangiocytes) identified 24 subpopulations, which were grouped into five functional states: hepatoblasts (expressing SPINK1, AFP, and the stemness marker LGR5), mature hepatocytes (hepatocyte1, ALB, SERPINA1, and CYP3A7), mature cholangiocytes (cholangiocyte1, KRT7, KRT19, and TACSTD2), and two transitional states coexpressing hepatoblast markers with hepatocyte or cholangiocyte markers (hepatocyte2 and cholangiocyte2) (Figures 4A and 4B). Pseudotime analysis revealed a bifurcated differentiation trajectory from hepatoblasts toward hepatocytes or cholangiocytes (Figure 4C). Hepatocyte differentiation was characterized by increased ALB, TTR, and SERPINA1, whereas cholangiocyte development involved the upregulation of KRT7, KRT19, and endothelial genes (KDR, CDH5), suggesting an endothelial influence on bile duct formation (Figures 4D and S4A). LGR5 expression decreased over time, reflecting the loss of stemness. Notably, only the embryo samples and the ASC-derived organoid of Blutt et al. included mature hepatocytes or cholangiocytes. Most PSC-derived organoids remained in transitional states, especially the vascularized organoid of Saiki et al., which retained a high proportion of undifferentiated hepatoblasts (Figure 4C).

A similar subpopulation analysis was performed for mesoderm-derived hepatic stellate cells and endothelial cells. By comparing the expression of lineage-specific marker genes, we identified a group of cells that highly expressed features of both lineages and showed significant enrichment of genes related to cell proliferation (Figures 4E and 4F). Previous studies have suggested that endothelial and stellate cells in the fetal liver may originate from a common progenitor population known as stellate-endothelial progenitors (SEpro).^29^ In our study, pseudotime analysis further supported this hypothesis, demonstrating that SEpro functions as a shared precursor capable of differentiating into both endothelial and stellate lineages. Moreover, the expression patterns of signature genes specific to each lineage diverged along the developmental trajectory, reflecting their lineage commitment during organoid maturation (Figures 4G and S4B).

### Bipotent differentiation potential of hepatoblasts

We compared the expression patterns of key transcription factors and signaling pathways involved in liver development across different organoid protocols to explore the underlying causes of the divergent hepatoblast differentiation outcomes (Figure 4H). The CEBPA gene encodes the C/EBPα protein, which binds to *cis*-regulatory elements of liver-specific genes and plays critical roles in hepatic energy metabolism, regeneration, and cytochrome P450 gene expression.^32^ Previous studies have shown that CEBPA can inhibit the differentiation of hepatoblasts into cholangiocytes.^33^ Correspondingly, in the CEBPA gene knockout mouse model, the expression of liver-specific transcription factors and bile duct transcription factors is unstable, resulting in significant maturity disorders in liver cells.^34^ In our analysis, CEBPA expression was absent in the organoids generated by Blutt et al. but was highly expressed in fetal liver samples and in the organoids from Velazquez et al., which tended to favor hepatocyte differentiation. Similarly, other transcription factors such as PROX1 and GATA6 were significantly expressed in hepatocyte-fated organoids. In contrast, genes involved in TGFβ and NOTCH signaling—critical regulators of cholangiocyte differentiation^35^^,^^36^—such as TGFB1/2, NOTCH1/2/3, JAG1, and LAMA5—were highly expressed in the Blutt et al. organoids. These findings suggest that the modulation of transcription factor expression and signaling activity may influence the fate decisions of hepatoblasts toward either hepatocytic or cholangiocytic lineages.

### Cell-cell communication analysis reveals regulatory signaling networks in liver organoids

**Figure 5:**
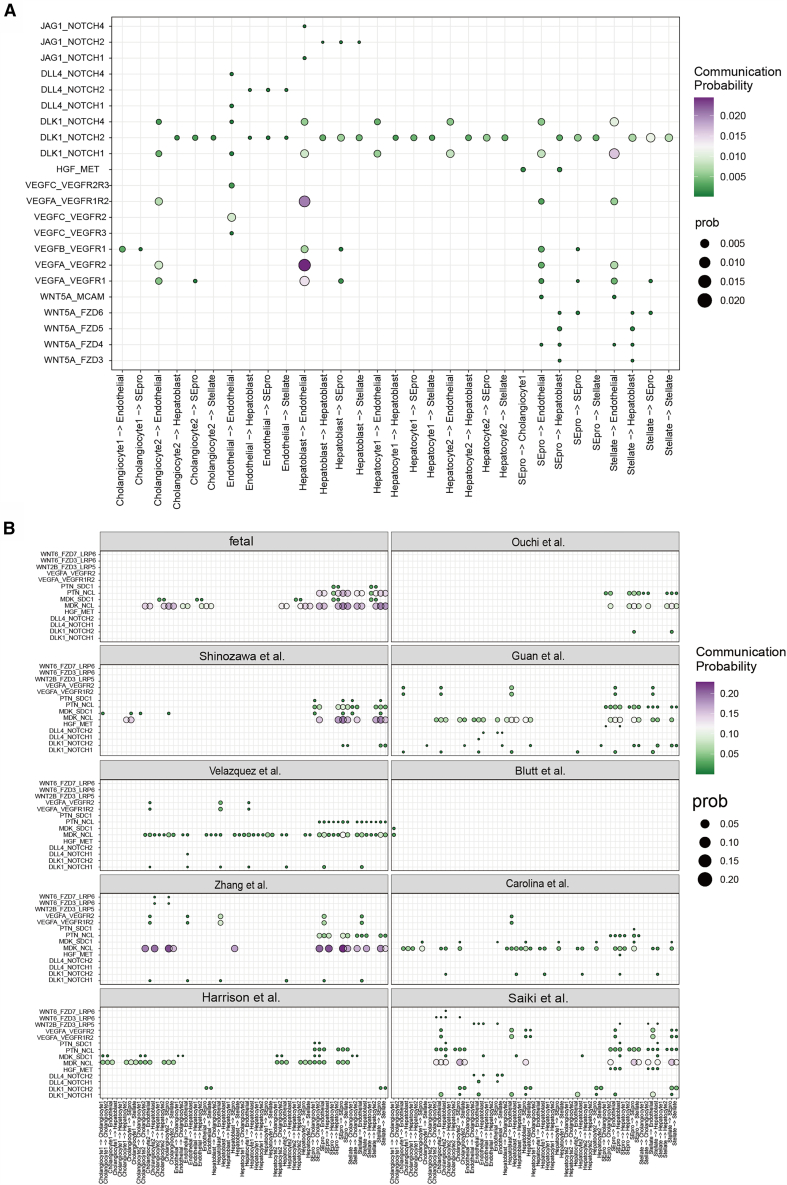
Cell-cell communication in liver organoids (A) Bubble diagram summarizes the inferred interaction strengths of major developmental signaling pathways, including NOTCH, HGF, VEGF, and WNT, among annotated cell types within liver organoids, as estimated from ligand-receptor expression patterns. (B) Comparison of cell-cell communication intensities mediated by selected ligand-receptor pairs across different organoid culture protocols.

Based on subpopulation analysis, we systematically examined cell-cell communication between mesoderm- and endoderm-derived cell types to explore potential interactions during liver development (Figure S5). As shown in Figure 5A, the NOTCH signaling pathway appears to play a central role in mediating cross-lineage communication. Previous studies have shown that DLL4, which is expressed by endothelial cells, interacts with DLK1/NOTCH2 on hepatoblasts and hepatocytes, suggesting that hepatoblasts help shape their vascular microenvironment. Conversely, endothelial cells may influence hepatoblast-to-cholangiocyte differentiation via NOTCH signaling.^36^ In line with these observations, our analyses indicate the presence of NOTCH signaling activity in liver organoids, particularly through DLK1–NOTCH2 interactions inferred across multiple cell types, and among these, the intercellular interactions mediated by DLK1-NOTCH2 were most evident in the organoids cultivated by Saiki et al. (Figure S6A). Additionally, JAG1–NOTCH signaling was observed between hepatoblasts and various cell types, suggesting their potential for cholangiocyte differentiation. We also observed VEGF signaling from hepatoblasts to endothelial cells, suggesting a role in vascular induction. In addition, HGF secreted by stellate-endothelial progenitors (SEpro) binds to MET receptors on hepatoblasts and cholangiocytes to regulate development. WNT signaling, which is derived primarily from stellate cells, may support hepatoblast self-renewal, which is consistent with previous findings.^29^

Notably, in fetal liver samples, these classical pathways (NOTCH, VEGF, HGF, and WNT) were not dominant (Figure 5B and S6B). Instead, communication is mediated mainly by the MDK/PTN-SDC/NCL axis, which involves pleiotrophin family growth factors (PTN, MK) known to regulate cell growth, differentiation, and migration.^37^ These findings suggest that integrating PTN family signaling with developmental cues such as NOTCH, VEGF, HGF, and WNT may enhance organoid maturation and fidelity.

## Discussion

Liver organoids, as an *in vitro* culture model, offer an effective approach for studying liver development, disease mechanisms, and drug screening. However, a major challenge in the field is determining the extent to which *in vitro* culture systems can recapitulate the structure and function of native liver tissue. A recent review discussing methods for comparing primary hepatocytes with in vitro-cultured hepatocytes highlighted that a comprehensive evaluation can be achieved using multiple techniques,^38^ including microscopic morphology, transcriptomics, proteomics, and so on. Previous studies using RNA-seq have systematically compared the transcriptomic similarity of various liver models to primary hepatocytes, revealing that no *in vitro* model fully reproduces the transcriptional features of the liver.^39^ Another study, based on metabolomic profiling, showed that hiPSC-derived hepatocytes exhibit a high degree of similarity to primary hepatocytes in lipid metabolic patterns.^40^ Nevertheless, a systematic comparison between the two at single-cell resolution is still lacking. In this study, using the OrganoidDB resource previously established, we integrated single-cell transcriptomic datasets to construct a liver organoid atlas and systematically compared the similarities and differences between organoids generated under different culture conditions and fetal liver tissues at the cellular level, thereby providing single-cell evidence for evaluating the *in vivo* mimicry of liver organoids.

Our analyses identified a lack of hematopoietic lineages in liver organoids. The fetal liver plays a crucial role in embryonic hematopoiesis. Hematopoietic stem and progenitor cells (HSPCs) migrate into the liver bud at approximately week 6 post-fertilization, where they expand and differentiate within the hematopoietic niche provided by the fetal liver, giving rise to major blood lineages, including erythroid, lymphoid, and myeloid cells. In addition to hematopoiesis, this process also promotes hepatocyte maturation during development. Consequently, establishing an *in vitro* hematopoietic niche during organoid culture could significantly increase the clinical relevance and application potential of liver organoids. Several studies have provided valuable insights into the cellular components of the fetal hematopoietic niche. For example, Nestin^+^ stromal cells near the portal vein have been identified as key supporters of hematopoietic liver cells during fetal development.^41^ To demonstrate the role of stromal cells in supporting HSPC expansion, researchers isolated fetal liver stromal cells and reported that the AFT024 monoclonal stromal cell line could sustain HSPCs for 5–7 weeks, offering a niche for their *ex vivo* expansion.^42^ Moreover, stromal cells freshly isolated from fetal liver have been shown to secrete angiopoietin-like proteins 2 and 3 (ANGPTL2/3), which promote HSPC proliferation.^43^ Another study highlighted the importance of the endothelial cell-selective adhesion molecule (ESAM), expressed in endothelial cells, in regulating erythropoiesis.^44^ These findings collectively suggest promising directions for engineering hematopoietic niches in liver organoids.

Our study also sought to explore the roles of key growth factors in culture protocols that influence lineage decisions during liver organoid development. For example, retinoic acid has been reported to play an important role in hepatic stellate cell differentiation. Previous studies have shown that its nuclear receptor transcription factor RXRA is involved not only in hepatocyte differentiation but also in maintaining the quiescent state of stellate cell; its ligand, 9-cis-retinoic acid (9CRA), can enhance the functional maturation of iPSC-derived hepatocytes while effectively suppressing the differentiation of iPSCs toward the stellate cell lineage.^45^ In addition, Activin A also exerts essential regulatory functions during liver organoid culture. Recent work has shown that Activin A acts as a key negative regulator of intrahepatic bile duct development, inhibiting cholangiocyte maturation and the formation of branched ductal structures.^46^ In contrast, EGF and FGF tend to promote cholangiocyte epithelial fate. Specifically, EGF can drive the differentiation of bipotent human liver organoids toward cholangiocyte epithelial cell,^47^ whereas FGF signaling maintains the undifferentiated state of multipotent progenitors in the extrahepatic duct region and prevents their premature differentiation into hepatic parenchymal lineage.^48^ Moreover, EGF and FGF together contribute to strengthening the epithelial barrier function of cholangiocyte.^49^ Collectively, this evidence suggests that precisely modulating the types, concentrations, and temporal combinations of specific growth factors in culture systems may enable the directed differentiation of liver organoids toward hepatocytes, cholangiocytes, or non-parenchymal cells such as stellate cells. Such strategies may provide useful guidance for constructing liver organoid models that are more complex and functionally complete.

### Limitations of the study

Although our study has produced a series of results, we recognize that transcriptomic data alone cannot fully capture the developmental features of organoids. Future work combining multi-omics approaches and biological experiments will be needed to further validate and explore these findings. Moreover, the scope of the datasets included in this study is limited, and the heterogeneity of the dataset may lead to potential biases. Expanding the dataset in future analyses will enhance the robustness of the results—for example, by incorporating additional ASC-derived liver organoids or including adult liver tissues as comparative references. In addition, applying more advanced methods for multi-sample integration to mitigate batch effects represents another important direction for further investigation. Overall, the liver organoid cell atlas we constructed provides fresh perspectives for evaluating organoid fidelity, optimizing culture protocols, and advancing applications in clinical drug screening.

## Resource availability

### Lead contact

Requests for additional information and resources should be addressed to the lead contact, Jianbo Pan (panjianbo@cqmu.edu.cn).

### Materials availability

This study did not generate new unique reagents.

### Data and code availability

• Data: This article does not report original data; all data used in this study are publicly available, and accession numbers are provided in Table S1.
• Code: This article does not report original code.
• Other items: Any additional information required to reanalyze the data reported in this article are available from the lead contact upon request.

## Acknowledgments

This work was supported by the Research Startup Funds of 10.13039/501100004374Chongqing Medical University, the project of the top-notch talent cultivation program for graduate students of 10.13039/501100004374Chongqing Medical University (No. BJRC202214), China. The computing work in this article was partly supported by the 10.13039/501100021520Supercomputing Center of 10.13039/501100004374Chongqing Medical University, China.

## Author contributions

J.P. conceived and planned the study and supervised the analyses. Q.M. conducted the analyses and wrote the first draft. X.Z. reviewed the integrity and plausibility of the data analysis. J.P. and Q.M. revised the article and were responsible for the integrity of data acquisition and statistical analyses. All authors agreed to submit the article, read and approved the final draft, and take full responsibility for its content, including the accuracy of the data and its statistical analysis.

## Declaration of interests

The authors declare no competing interests.

## Declaration of generative AI and AI-assisted technologies in the writing process

During the preparation of this work, the author(s) used ChatGPT to improve readability and language. After using this tool/service, the authors reviewed and edited the content as needed and take full responsibility for the content of the published article.

## STAR★Methods

### Key resources table

**Table:** 

REAGENT or RESOURCE	SOURCE	IDENTIFIER
**Deposited data**		

scRNA for liver organoid	https://www.ncbi.nlm.nih.gov/geo/query/acc.cgi?acc=GSE130075	GSE130075
scRNA for liver organoid	https://www.ncbi.nlm.nih.gov/geo/query/acc.cgi?acc=GSE141183	GSE141183
scRNA for liver organoid	https://www.ncbi.nlm.nih.gov/geo/query/acc.cgi?acc=GSE154883	GSE154883
scRNA for liver organoid	https://www.ncbi.nlm.nih.gov/geo/query/acc.cgi?acc=GSE159491	GSE159491
scRNA for liver organoid	https://www.ncbi.nlm.nih.gov/geo/query/acc.cgi?acc=GSE166589	GSE166589
scRNA for liver organoid	https://www.ncbi.nlm.nih.gov/geo/query/acc.cgi?acc=GSE188541	GSE188541
scRNA for liver organoid	https://www.ncbi.nlm.nih.gov/geo/query/acc.cgi?acc=GSE245379	GSE245379
scRNA for liver organoid	https://www.ncbi.nlm.nih.gov/geo/query/acc.cgi?acc=GSE240019	GSE240019
scRNA for liver organoid	https://www.ncbi.nlm.nih.gov/geo/query/acc.cgi?acc=GSE270807	GSE270807
scRNA for liver fetal	https://www.ebi.ac.uk/gxa/sc/experiments/E-CURD-98	E-CURD-98

**Software and algorithms**		

R (v4.1.0)	https://www.r-project.org/	–
Seurat(v4)	https://satijalab.org/seurat/	–
Monocle2	https://cole-trapnell-lab.github.io/monocle-release/	–
CellChat	https://github.com/jinworks/CellChat	–

### Experimental model and study participant details

In this study, we analyzed single-cell transcriptomic datasets of liver organoids generated by multiple laboratories, together with single-cell transcriptomic data from human fetal liver tissues spanning 5–19 weeks of gestation.

### Method details

#### Data sources

As shown in Table S1, liver organoid samples were curated from the previously developed OrganoidDB transcriptomic database on the basis of the following inclusion criteria: (1) single-cell RNA-seq data generated using 10X Genomics platforms; (2) availability of corresponding published studies to ensure access to detailed culture protocols (e.g., growth factors, timepoints); (3) sample annotation as “Liver” in the OrganoidDB “Organ” field and “Homo sapiens” in the “Organism” filed; and (4) exclusion of samples subjected to drug treatment or genetic modification to avoid confounding effects on cellular states. Additionally, three published datasets involving vascularized liver organoids were incorporated to assess the impact of vascularization on organoid development. To evaluate the developmental fidelity of liver organoids, a human fetal liver single-cell dataset (E-CURD-98, based on 10X genomics) comprising samples from 5‒19 weeks of gestation was included as a developmental ref.^27^

#### Data processing and analysis

In this study, we utilized the Seurat (v4.1.1) package^50^ for data preprocessing and analysis. During the creation of the Seurat object, low-expression genes expressed in fewer than 3 cells were filtered out by setting the *min.cells* parameter in the *CreateSeuratObject* function. In addition to filtering low-expression genes, a series of thresholds were applied to remove low-quality cells. Specifically, cells with detected gene counts ranging between 200 and 4000 were retained to filter out cell debris and most doublets. Furthermore, cells with mitochondrial gene proportions exceeding 20% were filtered out to ensure cell viability. The normalization of the expression matrix followed the *NormalizeData*, *FindVariableFeatures* and *ScaleData* workflow. The *NormalizeData* function was used to normalize the data using the log normalization method (“*LogNormalize*”) with a scale factor of 10,000; the *FindVariableFeatures* function employed variance-stabilizing transformation (VST) to select the top 2000 highly variable genes; subsequently, the *ScaleData* function was applied to normalize the data on the basis of these highly variable genes.

Owing to differences in experimental conditions, library preparation, and sequencing across samples, nonbiological batch effects may obscure true biological differences between samples. To address this, the Harmony (v0.1.0) package^51^ was used to remove batch effects during the integration of preprocessed samples. Dimensionality reduction analysis of the integrated dataset was performed using the *RunTSNE* and *RunUMAP* functions from the Seurat package. The *FindNeighbors* and *FindClusters* functions were used for cell clustering. In this study, the resolution parameter in the *FindClusters* function was set to 0.8 to obtain more granular cell clusters, facilitating more detailed cell annotation.

#### Cell type annotation

Accurately identifying and annotating cell subpopulations within samples is crucial in single-cell transcriptomic analysis. In this study, we identified cell subpopulations on the basis of known cell type-specific marker genes and combined enrichment analysis results to define these cells. Specifically, the *FindAllMarkers* function from the Seurat package was used to calculate differentially expressed genes for each cell subpopulation in the integrated dataset. We screened for marker genes that met the criteria of *avg_log2FC* > 0.25 and *p_val_adj* < 0.05. These identified marker genes were then compared with cell type-specific gene sets collected from authoritative databases such as CellMarker^52^ and PanglaoDB,^53^ alongside commonly used liver tissue cell markers from published studies, including hepatocyte markers (e.g., *ALB* and *CYP3A4*), cholangiocyte markers (e.g., *KRT7* and *KRT19*), and hepatic stellate cell markers (e.g., *ACTA2* and *PDGFRB*), to facilitate preliminary classification of cell types. To increase annotation reliability, we further utilized the clusterProfiler package^54^ to perform Gene Ontology (GO) biological process enrichment analyses on the marker genes of each subpopulation, identifying significant associations with biological functions. By integrating the expression characteristics of marker genes and the results of pathway enrichment analyses, we systematically named the cell subpopulations. This comprehensive approach ensures accurate and reliable annotation of cell types within liver organoid models, facilitating deeper insights into their functional characteristics and developmental processes.

#### Cell‒cell communication analysis

Cell–cell communication analysis of the integrated dataset was conducted using the CellChat package^55^ (v1.5.0). First, the normalized expression matrix and cell type annotations were extracted from the Seurat object and used to construct a CellChat object via the *createCellChat* function. Next, highly expressed genes in each cell subtype were identified using the *identifyOverExpressedGenes* function. These genes were further used to infer significantly active ligand–receptor pairs through the *identifyOverExpressedInteractions* function. The communication probabilities between cell populations were computed using the *computeCommunProb* function with the parameter set to *type = ‘triMean’*, which calculates the average gene expression within cell groups using the trimmed mean method to increase the sensitivity in detecting strong interaction signals. Finally, high-confidence interactions (p < 0.05) were selected, and significantly enriched signaling pathways were identified.

#### Pseudotime analysis

Pseudotime analysis is a commonly used technique in single-cell transcriptomic studies that infers the continuous progression of cellular states on the basis of gene expression profiles. It enables the reconstruction of developmental trajectories by mapping cells onto a branched structure that simulates dynamic biological processes. In this study, we performed pseudotime analysis on a liver organoid single-cell dataset using the Monocle2 package^56^ (v2.22.0) to reconstruct the developmental and differentiation trajectories of liver cells. To improve computational efficiency, we first extracted a subset of the original dataset. Next, we used the *differentialGeneTest* function to assess gene expression dispersion and selected the top 1,000 genes with the highest variability as the feature genes for trajectory inference. This approach enables the identification of key regulatory factors that exhibit significant expression fluctuations during cell state transitions. Finally, dimensionality reduction was performed with the “DDRTree” algorithm, which projects cells from high-dimensional expression space into a low-dimensional trajectory space and constructs a branched developmental trajectory tree.

#### Comparative analysis of liver organoids and the fetal liver

To assess the similarity between liver organoids and fetal liver tissues at the transcriptomic level, we conducted a multidimensional comparative analysis. Using the *AverageExpression* function from the Seurat package, we computed the average expression of each gene within individual cell clusters. On the basis of these average expression values, we calculated Pearson correlation coefficients to evaluate the transcriptional similarity between each organoid culture protocol and fetal liver samples across different cell subpopulations. In addition, we identified DEGs between each organoid culture protocol and fetal liver tissue, selecting significant genes with adjusted p values < 0.05 and |avg_log2FC| > 1. These DEGs were subjected to GO and KEGG pathway enrichment analyses to explore functional differences. Furthermore, to investigate global transcriptional differences between organoids and fetal livers as a whole, we performed GSEA to identify pathways that were differentially enriched between organoid and fetal samples. In addition, in order to quantify the differences in signaling pathways between organoids and fetal samples, we obtained the gene sets related to specific pathways from the KEGG database, and used the *AddModuleScore* function of the Seurat package to calculate the scores of these specific pathways in different samples.

## Supplemental information

Document S1. Figures S1–S6

Table S1. Inclusion Criteria and metadata of Datasets
