# Supplementary material for: A cell atlas of multiple liver organoids and the fetal liver based on scRNA-seq
Source: iScience. 2026 Feb 7;29(3):114955. doi: 10.1016/j.isci.2026.114955 (PMC12927097; doi:10.1016/j.isci.2026.114955)
Supplement: Document S1. Figures S1–S6 [file mmc1.pdf]

iScience, Volume *29*

Supplemental information

A cell atlas of multiple liver organoids and the fetal liver based on scRNA-seq

Qinfeng Ma, Xu Zhang, and Jianbo Pan

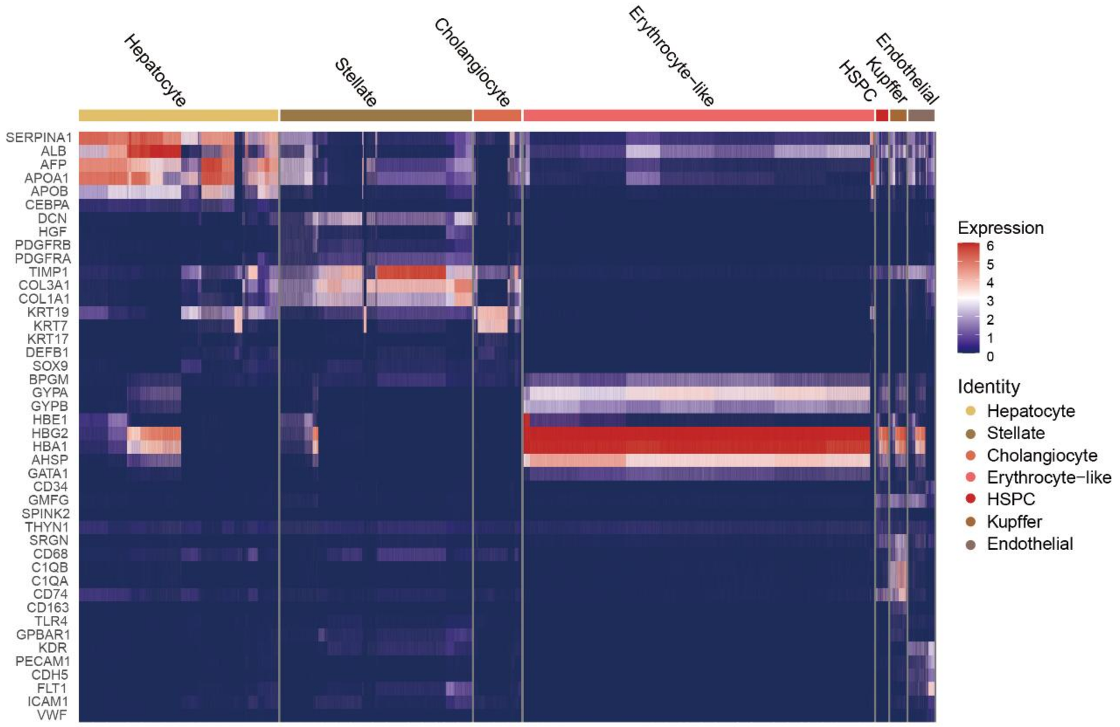

Figure S1. The heatmap displays representative marker genes for the major annotated cell types. Rows correspond to selected marker genes, and columns represent individual cell. Color intensity reflects relative gene expression levels. The color bars above the heatmap indicate the corresponding cell type annotations.

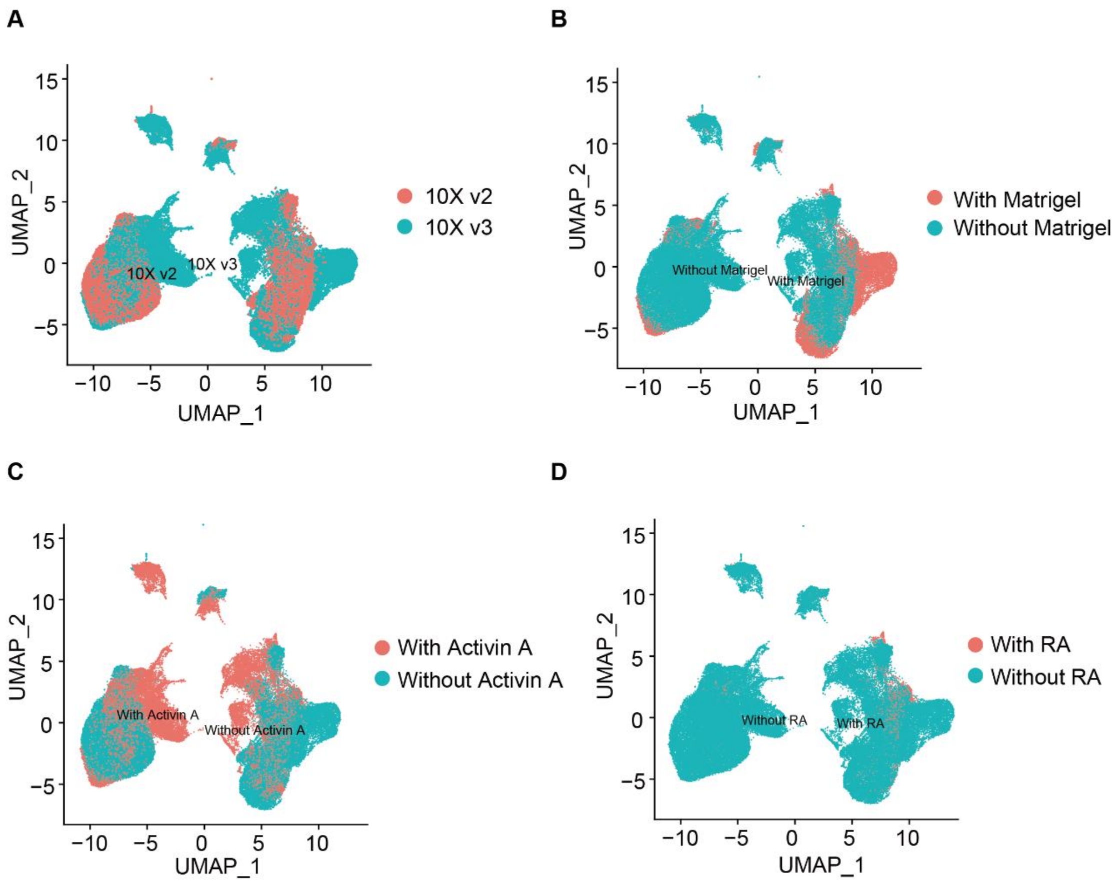

Figure S2. UMAP plot colored by (A)sequencing strategy, (B) with Matrigel or not, (C) with Activin A or not, (D) with retinoic acid or not. In panel A, red color indicates the 10X v2 version which includes samples from Velazquez et al. and Harrison et al, while blue color represents the 10X v3 version which contains organoids from Guan et al., Blutt et al., Carolina et al., and Saiki et al.

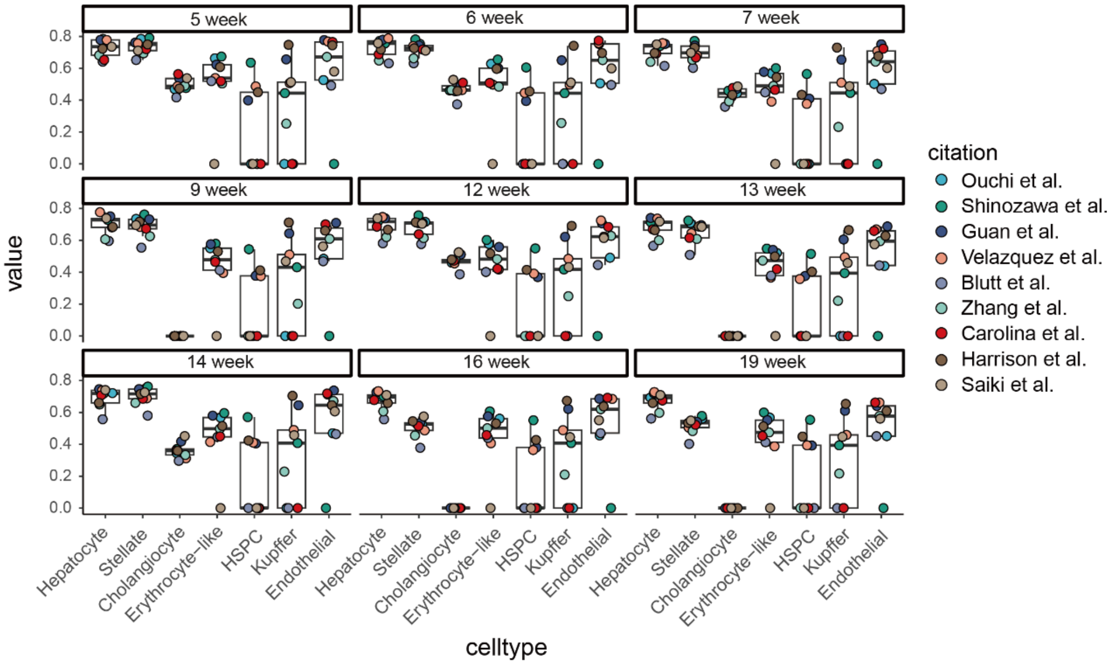

Figure S3. Box plots showing the transcriptome correlation between the organoid of different protocols and the fetal sample at various development time points. The x-axis represents different cell types, the y-axis represents the correlation coefficient, and the protocols are filled in with different colors.

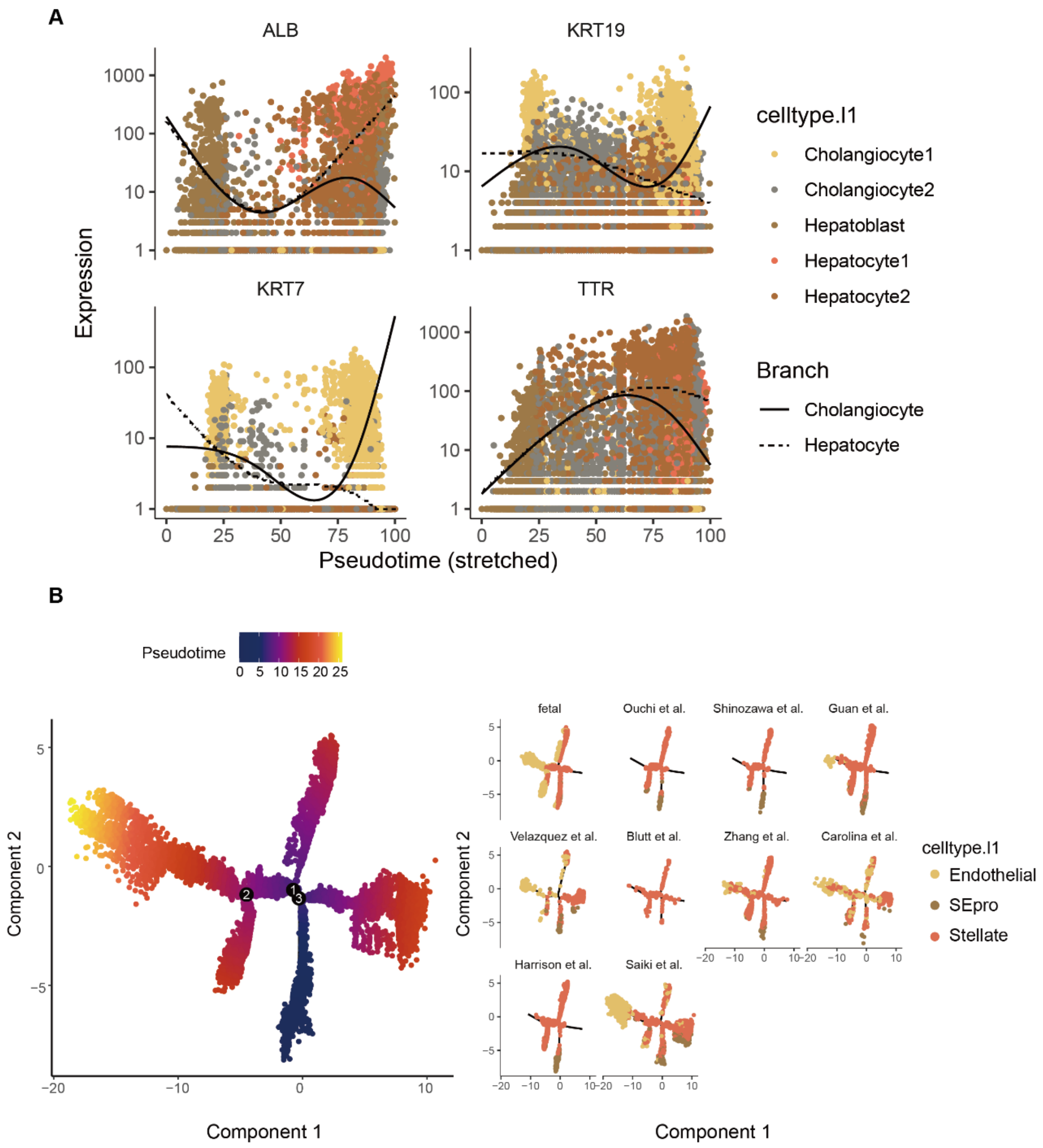

Figure S4. (A) Expression dynamics of hepatocyte and cholangiocyte markers along the developmental trajectory. (B) Developmental trajectory analysis of mesoderm lineage differentiation. The left figure illustrates the inference of a pseudotime-based developmental trajectory from single-cell transcriptomics data, and the right figure shows a comparison of the differentiation trajectories of different organoid cultivation schemes, with cells colored according to the cell types.

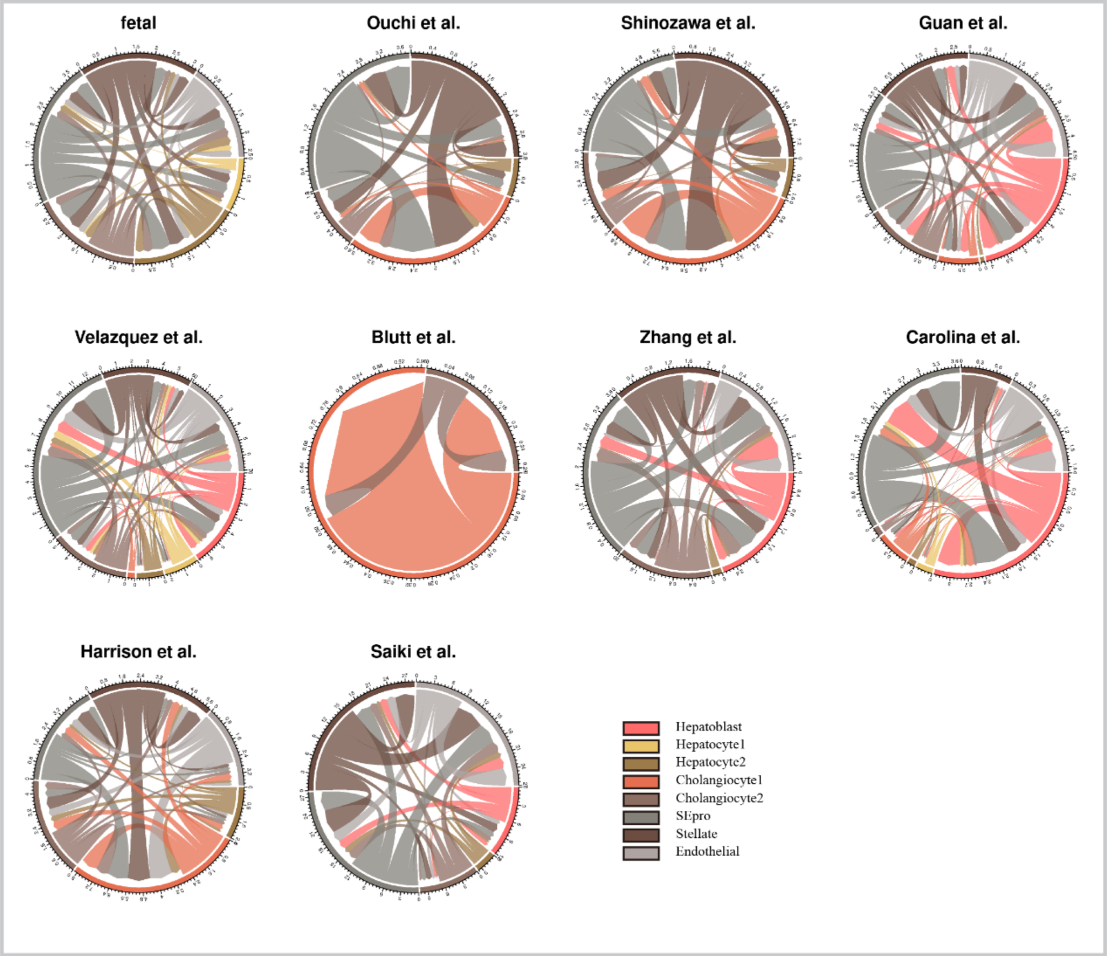

Figure S5. Chord diagram illustrating intercellular communication patterns. Different colors represent distinct cell types, and the thickness of the arrows indicates the strength of the interaction.

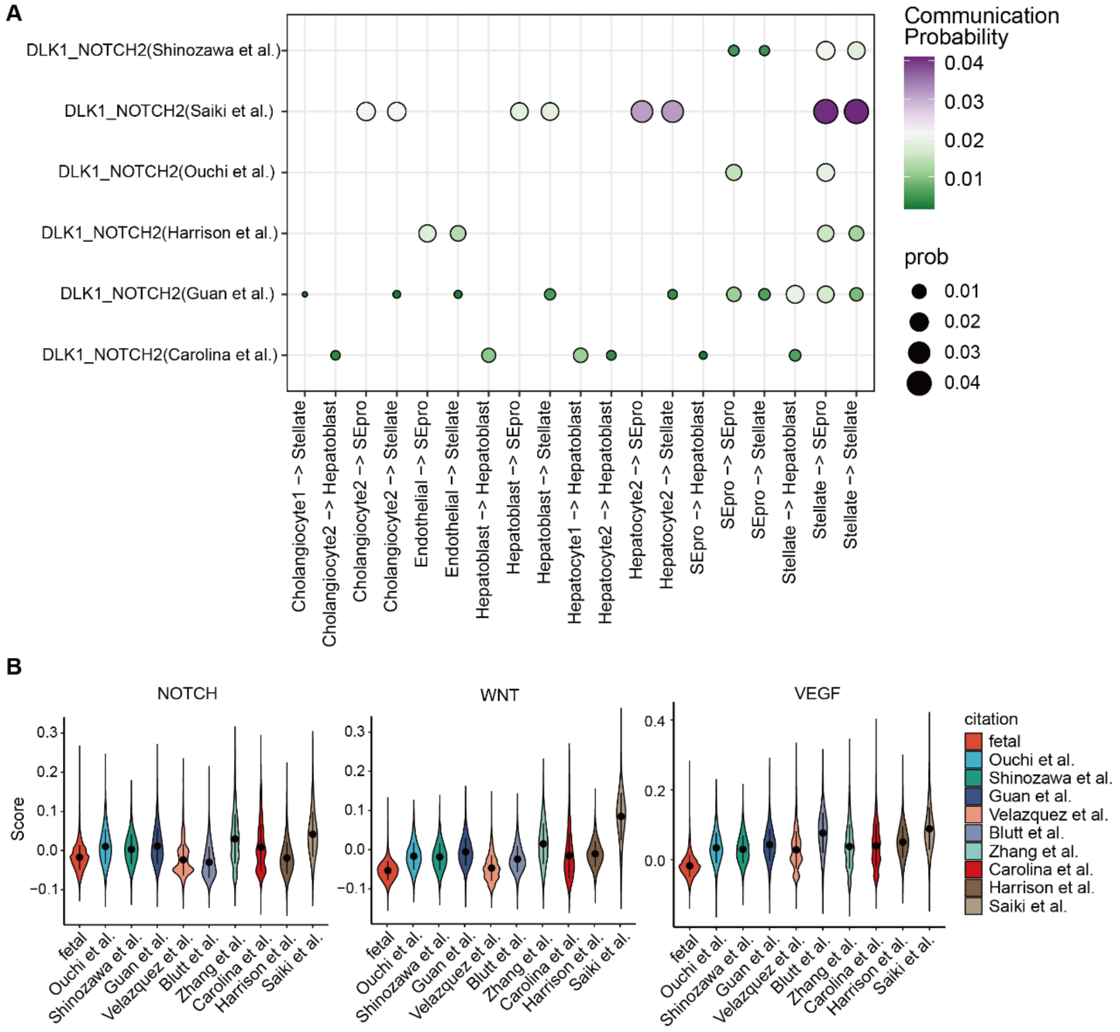

Figure S6. (A) Inferred probability of cell–cell communication mediated by the DLK1–NOTCH2 ligand–receptor pair across different liver organoid culture protocols. (B) Violin plots showing the distribution of signaling pathway activity scores for NOTCH, WNT, and VEGF pathways in fetal liver samples and organoids from various culture protocols. Different colors represent the sample origin, allowing comparison of pathway activity across fetal and organoid conditions.
